# The Interpretation of E-Motions in Faces and Bodies Derived from Static Artworks by Individuals with High Functioning Autistic Spectrum

**DOI:** 10.3390/vision5020017

**Published:** 2021-03-25

**Authors:** Maria Elisa Della-Torre, Daniele Zavagno, Rossana Actis-Grosso

**Affiliations:** Department of Psychology, Università di Milano-Bicocca, 20126 Milano, Italy; m.dellatorre7@campus.unimib.it (M.E.D.-T.); daniele.zavagno@unimib.it (D.Z.)

**Keywords:** autism-spectrum-disorder, emotions, visual-arts

## Abstract

E-motions are defined as those affective states the expressions of which—conveyed either by static faces or body posture—embody a dynamic component and, consequently, convey a higher sense of dynamicity than other emotional expressions. An experiment is presented, aimed at testing whether e-motions are perceived as such also by individuals with autism spectrum disorders (ASDs), which have been associated with impairments in emotion recognition and in motion perception. To this aim we replicate with ASD individuals a study, originally conducted with typically developed individuals (TDs), in which we showed to both ASD and TD participants 14 bodiless heads and 14 headless bodies taken from eleven static artworks and four drawings. The Experiment was divided into two sessions. In Session 1 participants were asked to freely associate each stimulus to an emotion or an affective state (Task 1, option A); if they were unable to find a specific emotion, the experimenter showed them a list of eight possible emotions (words) and asked them to choose one from such list, that best described the affective state portrayed in the image (Task 1, option B). After their choice, they were asked to rate the intensity of the perceived emotion on a seven point Likert scale (Task 2). In Session 2 participants were requested to evaluate the degree of dynamicity conveyed by each stimulus on a 7 point Likert scale. Results showed that ASDs and TDs shared a similar range of verbal expressions defining emotions; however, ASDs (i) showed an impairment in the ability to spontaneously assign an emotion to a headless body, and (ii) they more frequently used terms denoting negative emotions (for both faces and bodies) as compared to neutral emotions, which in turn were more frequently used by TDs. No difference emerged between the two groups for positive emotions, with happiness being the emotion better recognized in both faces and in bodies. Although overall there are no significant differences between the two groups with respect to the emotions assigned to the images and the degree of perceived dynamicity, the interaction Artwork x Group showed that for some images ASDs assigned a different value than TDs to perceived dynamicity. Moreover, two images were interpreted by ASDs as conveying completely different emotions than those perceived by TDs. Results are discussed in light of the ability of ASDs to resolve ambiguity, and of possible different cognitive styles characterizing the aesthetical/emotional experience.

## 1. Introduction

The present study is part of larger project focusing on the link between motion perception and the perception and recognition of emotions, conveyed by both faces and bodies. Studies on the expression of emotions have been mainly focused on emotional faces (e.g., [1,2,3,4]); however, it is now generally accepted that body language or “bodily kinematics” is equally important in conveying emotionally relevant information. The link connecting emotions and motion is thus becoming clearer, with a growing number of studies focused on the kinematic pattern specific for each emotion (e.g., [5,6,7]), and consequently there has been an increase in the employment of dynamic displays as stimuli—such as, for example, the Johansson’s [8,9] point-light biological motion display (PLD), with actors expressing different emotions—instead of the classic photographs portraying emotional faces (e.g., Radboud Faces Database, [10]).

A specific part of the project (i.e., “E-motion in the Visual Art”, [11,12,13]) is dedicated to the visual arts. In particular, we hypothesize that some emotions—e.g., anger, fear—are expressed in a way that is more “motion related” as compared to the expression of other affective states (such as, for example, puzzlement). We referred to these emotions as “e-motions” [14] to emphasize the relevant presence of a motion (or dynamic) component in their expression. Following this reasoning, we hypothesize that the static representations of both faces and bodies expressing these specific emotions embody a stronger sense of motion and, consequently, should convey a greater sense of dynamicity. According to our hypothesis, there is a mutual relationship between motion and emotion: if on the one hand, motion per se can improve the recognition of emotions, as has been demonstrated (e.g., [5,6,7]), then also the opposite is true: emotions may contribute to the perception of motion or dynamism within static representations of the human figure. Thus, the representation of a human figure expressing a specific e-motion should appear more dynamic, and therefore could be (or have been) employed by visual artists to enhance the representation of motion or dynamicity in static artworks.

In a previous work by our group [11], this hypothesis was preliminary tested by asking participants to classify human figures taken from artworks according to (a) the type of emotion depicted (from a given set of eight possible emotions) and to rate (b) the dynamicity conveyed by the emotion depicted. Classifications and ratings were conducted separately for the body (i.e., headless bodies) and the face (i.e., bodiless heads) of the depicted human figure digitally isolated from the context of their artwork. Results confirmed that depicted emotions could be distinguished on a dynamicity scale. In particular, specific emotions, such as anger, fear, or disgust, appear to incorporate a higher sense of dynamicity (i.e., e-motions). In addition, it was found that faces were scored as less dynamic than their corresponding bodies. Actis-Grosso and Zavagno [11] is thus the first evidence that static emotional faces and bodies in the visual arts could be implicitly dynamic, allowing the observer to extract dynamic information from their static representation.

In the study presented here we are interested in seeing whether e-motions are perceived as such also by individuals with autism spectrum disorders (ASDs). In particular, we hypothesize that it would be possible to find a specific impairment for ASDs in recognizing “dynamic” emotions in static pictures. The reason why we expect to find this difference between ASDs and typically developed (TD) individuals is based on previous findings on both emotion recognition and motion perception in ASDs, as better detailed below.

Emotion processing and emotion recognition have been widely investigated in ASD individuals, who have impairments in social cognition. In particular, researchers studying the processing of emotional information in ASD have also identified difficulties in recognizing emotions through various stimuli, such as faces and point-light biological motion displays (PLDs, e.g., [15,16,17]). In addition, ASDs exhibit hypersensitivity to local elements of the visual input and, more generally, they exhibit an elevated motion coherence threshold [18], which implies that for these individuals it is more difficult to perceive, for example, a human being when presented as a moving PLD [19]. This impairment is consistent with the weak central coherence theory (WCC) [20], which posits that people with autism have an inherent bias towards processing parts of stimuli and an inability to integrate these into a Gestalt. This bias becomes crucial when local elements should be integrated in a short temporal interval—as in the recognition of emotions from body movements [18]—in order to extract global meaningful information. Supporting this theory, difficulties in recognizing “emotional” PLDs have been consistently reported in people with ASD [18,19,21,22,23,24,25,26,27], with contradictory results when ASDs are asked to recognize non-emotional actions from PLDs [17,23,25,28,29]. Thus, the WCC theory could account for the ASD’s difficulty in processing the emotional content of body movements, but it is still not clear whether and how the theory applies to simple actions.

In a study aimed at comparing a population of typically developed (TD) adults with individuals with high level of autistic traits (HAT) in the recognition of emotions both through static faces and PLDs [21], we found for TDs an advantage for motion kinematics in the recognition of fear, which was not present in HAT participants. Furthermore, in TD participants an advantage was found for the recognition of happiness, both for faces (i.e., happy face advantage, [30,31]) and for bodies (i.e., happy body advantage, noticed for the first time in [21]), whereas HAT individuals did not show significant advantages for any emotion. Other studies report a specific impairment in the recognition of negative emotions (such as disgust, anger, sadness, and fear, [26,32,33]), with several studies reporting a specific impairment for fear [34,35,36,37,38]. Furthermore, in a study by Philip et al. [26] a general difficulty for ASDs was found in recognizing emotions from static body posture, with a worst performance for fear and anger [39].

Relying on these findings we decided to replicate the study in [11] with an ASD population, to investigate whether the differences between TDs and ASDs in the perception and recognition of emotions would be present also when the emotions are portrayed in an artwork and could thus ultimately influence ASDs appreciation of the visual arts. In particular, for emotional faces we hypothesize a difference between ASDs and TDs in recognizing negative emotions and neutral affective states, while we expect similar results for positive emotions. For emotional bodies we expect to find instead a worst performance for ASDs in recognizing body emotion, due not only to their difficulty in emotion recognition, but also because of their impairment in the recognition of bodily kinematics. The emotions conveyed by bodies are usually associated to motion in real life, thus we might expect that, when asked to associate an emotion to a static body, participants rely on static signals associated to implied motion (such as unstable postures representing a transition from state A to state B, see [40,41]), a task that should be particularly difficult for ASDs. This difficulty would also result in a difference between the two groups with regards to the correspondence between the emotion attributed to the face and the corresponding body emotion. Regarding the degree of dynamicity attributed to each emotion (as conveyed by both face and body), for both groups we expect a higher degree of dynamicity attributed to bodies as compared to faces, with a possible difference between groups, because of ASDs’ difficulty in processing motion information. This difficulty could lead them to attribute a different degree of inherent dynamicity to a static body. We do not have a specific hypothesis about the direction of this expected difference.

To this aim we firstly ran a pilot study with only two ASD participants, to see whether stimuli and procedure from the original study were applicable also to ASD individuals. From the pilot we derived the necessity to slightly modify the original set of stimuli and the procedure, as described in the following sections. Given this change in stimuli and procedure, results from ASDs were compared with a new group of TD participants, who underwent the same procedure as the ASDs.

The most important change in the procedure was that participants were firstly asked to freely report the emotion conveyed by each stimulus. If they were unable to answer, then the experimenter showed them a list of eight possible emotions (the same as in [11]). This change in the procedure, mainly aimed to avoid confounding ASD individuals with too many possible emotions associable to each stimulus, also allowed us to observe the range of verbal expressions defining emotions between the two groups. We expected to observe a narrower range of verbal expressions defining emotions in ASDs, in line with [42], where ASDs showed a description of emotions mainly based on standard definitions, without any reference to personal experiences. Furthermore, several studies asking ASD individuals to verbally recognize affective states show a general impairment in recognizing neutral affective states in emotional faces (i.e., corresponding to an expression that is neither negative nor positive, such as boredom, interest, or disagreement [43,44,45,46]), with an interesting study demonstrating that this impairment is present only in verbal recognition and not in matching two static body postures expressing the same affective states [47]. We could thus hypothesize an additional difference in the recognition of neutral affective states, with ASDs’ performance being worse.

## 2. Method

### 2.1. Participants

Forty-five individuals took part in the experiment: 21 typically-developed participants (TD) (18–57 years old; mean age: 27.57, SD: 13.02; female: 16) and 24 participants with Autistic Spectrum Disorder (ASD, 20–54 years old; mean age: 32.21, SD: 10.56; female: 1). All participants had normal or corrected-to-normal vision, were unaware of the purpose of the study and participated to the experiment as unpaid volunteers.

Participants with ASD were recruited from three no-profit organizations. They were diagnosed from different clinical teams as follows: 13 participants diagnosed with Asperger Syndrome (AS) according to DSM-TR-IV and ICD-10 criteria and 11 diagnosed with Autism Spectrum Disorder, according to DSM-V criteria. Reliable IQ measures for 17 AS participants were obtained (mean IQ: 121.059, SD: 15.08) by the same clinical teams who made the ASD diagnosis.

### 2.2. Stimuli

**Figure 1:**
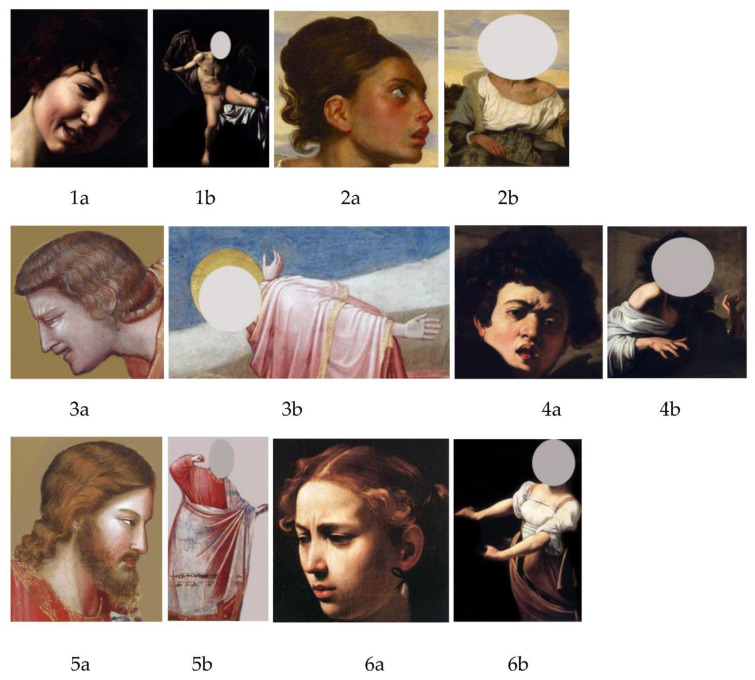

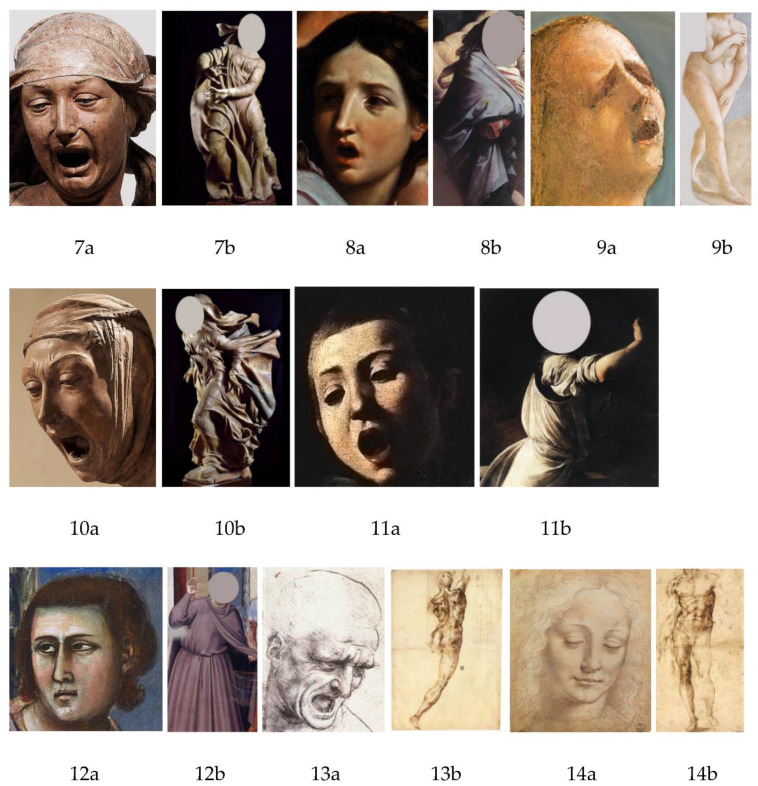
Stimuli used in the experiment. **1.** Head (**a**) and body (**b**) taken from Michelangelo Merisi da Caravaggio, *Amor vincit omnia*, 1602–1603, Berlin, Staatliche Museen. **2.** Head (**a**) and body (**b**) taken from Eugène Delacroix, *Orphan girl at the cemetery*, 1824, Paris, Louvre Museum. **3.** Head (**a**) and body (**b**) taken from Giotto, *The mourning of Christ*, 1303–1305, Padua, Scrovegni Chappel **4.** Head (**a**) and body (**b**) taken from Michelangelo Merisi da Caravaggio, *Boy bitten by a lizard*, 1595–1596, Florence, Longhi Foundation. **5.** Head (**a**) and body (**b**) taken from Giotto, *The expulsion of merchants from the temple*, 1303–1305, Padua, Scrovegni Chappel. **6.** Head (**a**) and body (**b**) taken from Michelangelo Merisi da Caravaggio, *Judith and Holofernes*, 1602, Rome, Barberini Palace **7.** Head (**a**) and body (**b**) taken from Nicolò dell’Arca, *Lamentation (The mourning of Christ),* particulars of Maria from Cleofa, 1463–1490, Bologna, Church of Santa Maria della Vita. **8.** Head (**a**) and body (**b**) taken from Guido Reni, *Massacre of the innocents*, 1611, Bologna, Pinacoteca Nazionale. **9.** Head (**a**) and body (**b**) taken from Giotto, *The expulsion from the garden of Eden*, particulars of Eve, 1424–1425, Firenze, Brancacci Chappel. **10.** Head (**a**) and body (**b**) taken from Nicolò dell’Arca, *Lamentation (The mourning of Christ),* particulars of Maria Maddalena, 1463–1490, Bologna, Church of Santa Maria della Vita. **11.** Head (**a**) and body (**b**) taken from Michelangelo Merisi da Caravaggio, *Martyrdom of Saint Matthew*, 1600–1601, Rome, Church of San Luigi dei Francesi. **12.** Head (**a**) and body (**b**) taken from Giotto, *The expulsion of merchants from the temple*, 1303–1305, Padua, Scrovegni Chappel. **13a.** Leonardo Da Vinci, *Studies for a head*, 1504–1505, Budapest, Szépmuvészeti. **13b.** Michelangelo Buonarroti, *Nude male, seen from behind*, 1504, Florence, Casa Buonarroti. **14a.** Leonardo Da Vinci, *Head of a young woman*, 1504–1505, Firenze, Galleria degli Uffizi. **14b.** Michelangelo Buonarroti, *Nude male,* 1501–1502, Paris, Louvre Museum.

Stimuli were derived from the same eleven static artworks and four drawings used in [11]. From these artworks two set of stimuli were produced: fourteen bodiless heads (Set A) and fourteen headless bodies (Set B). Figure 1 shows the stimuli employed (they will be referred to the corresponding numbers throughout the paper): as it can be seen, in the last four images (13a and 13b; 14a and 14b) headless bodies and bodiless heads are not taken from the same artworks. These last stimuli served as a control for our hypothesis, with two emotional faces showing a different degree of inherent dynamism, and two bodies that were conceived by Michelangelo as anatomical studies. Slight modifications were done to the original stimuli, in order to make them suitable for ASD participants. To focus participants’ attention only on the facial expression or body posture, images were manipulated with Adobe Photoshop CS5^®^ to remove from the backgrounds elements that could be possible distractors, given (i) the well-known local-based perception in high functioning autism (rather than global-based perception, e.g., [48,49]) and (ii) the fact that ASD people tend to focus their attention on different details rather than facial expressions [50]. Thus, for example, in Caravaggio’s painting *Judith and Holofernes*, we deleted Holofernes’ head (which was originally in the hands of Judith), as it is shown in 6b.

### 2.3. Procedure

The stimuli were displayed on a 7-inch PLS LCD display (Samsung Galaxy Tab II^®^; Resolution: 600 × 1024 pixels). Each participant sat at a comfortable viewing distance from the screen in a dimly lit room. Stimuli presentation was controlled by the experimenter.

The experiment was divided into two sessions. Session 1 was aimed at testing the emotion associated to each stimulus, whereas session 2 was focused on perceived dynamicity. Participants were asked to rate both emotions and dynamicity on a 7-point Likert scale (1 = not at all; 7 = very high). In each session they were tested at first on bodiless heads (Set A) and then on headless bodies (Set B). Instructions were provided verbally, and participants’ responses were separately recorded by the experimenter.

**Session 1 Emotions.** In the first session participants were asked to freely associate each stimulus to an emotion or an affective state (Task 1, option A) and then to rate its intensity on a 7-point Likert scale (Task 2). If they were unable to identify a specific emotion in Task 1, the experimenter showed a list of 8 possible emotions (i.e., joy, fear, anger, sadness, disgust, surprise, serenity, and puzzlement, the same as in [11]), and asked them to choose the emotion that they found best described the affective state represented by the stimulus (Task 1, option B). 

**Session 2 Dynamicity.** In the second session participants had to rate the perceived dynamicity conveyed by each image on a 7-point Likert scale.

Before starting, each participant was asked to describe the concept of dynamicity, in order to be sure that they were all responding to the same experimental question.

Finally, the TD group was asked to perform the Aspie Quiz^®^ [51], to verify whether they could possibly fit within the ASD group. The Aspie Quiz is a purely indicative test (without any diagnostic value), which provides a score to “neurodiverse/neurotypical traits” in adults, ranging from 0 to 200. The score can be used to give a reliable indication of autism spectrum traits prior to an eventual diagnosis. All TDs had a final score that falls within the neurotypical range (mean: 51.38, SD: 23.85).

All participants gave a written informed consent before testing. The study was conducted in accordance with the ethical standards laid down in the 1964 Declaration of Helsinki and fulfilled the ethical standard procedure recommended by the Italian Association of Psychology (AIP). The study was specifically approved by the local Ethics Committee of the University of Milano-Bicocca.

## 3. Results

### 3.1. Emotions

#### 3.1.1. Preliminary Classification of Emotions

Raw data for emotions consisted in two lists. (i) A first list of emotions (list A) that were freely identified by the participants in task 1, option A. Notice that list A had some missing values (when participants were not able to associate an emotion to a stimulus). (ii) A second list of emotions that participants selected from the list of eight terms provided when they were not able to freely indicate an affective term for a specific stimulus. This list was used to fill the missing values in list A, obtaining in this way a list (list B), in which every stimulus had an associated emotion.

We then ran separate analyses on those data. In particular, spontaneous identification of emotions (Task 1, option A) was firstly investigated to (a) identify a possible difference between groups and (b) to verify whether there were facial expressions and body postures that resulted as more difficult to decode. We then used list B to test for possible differences between stimuli (i.e., headless bodies vs. bodiless heads from the same artwork). After this analysis, we decided not to analyze (at least not at this stage) data obtained with Task 2 (i.e., ratings for perceived intensity of emotions), given the high number of missing values from ASDs in list A (as better detailed below), which raises some methodological concerns about the legitimacy of such an analysis (and consequent interpretation).

#### 3.1.2. Spontaneous Identification of Emotions: ASD vs. TD

**Figure 2:**
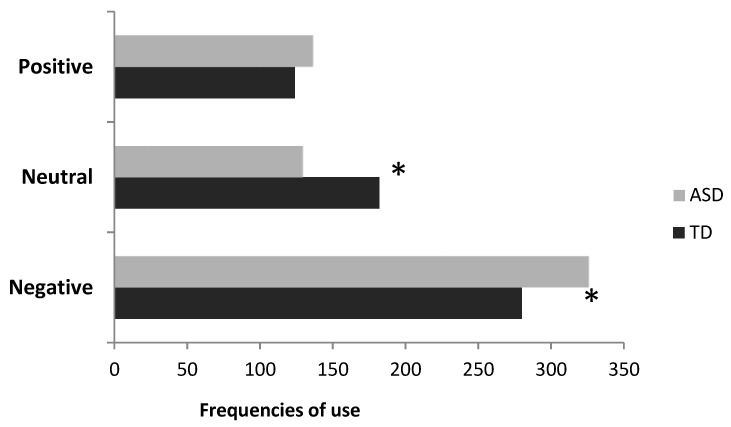
Frequency of use of terms corresponding to a neutral, a negative or a positive affective state distinguished by autism spectrum disorders (ASD) and TD participants. Asterisks represent statistically significant effects.

Altogether 98 different terms were spontaneously employed by participants to denote the affective states they identified as depicted in the stimuli. All the listed emotions and affective states were classified in three main categories: (1) words denoting emotions or affective states with a positive value (e.g., joy); (2) words denoting emotions or affective states with a neutral value (e.g., puzzlement); (3) words denoting emotions or affective states with a negative value (e.g., sadness). Overall, there was no difference between the two groups: TDs and ASDs used approximately the same number of words for positive (13 vs. 15 for TD and ASD, respectively), negative (33 vs. 31 for TD and ASD, respectively) and neutral (22 vs. 27 for TD and ASD, respectively, χ^2^ (2) = 0.53, *p* > 0.05) affective states. However, if we consider the frequency in which TDs and ASDs refer to a stimulus as expressing emotions with a different value (i.e., positive or negative), a difference emerged (χ^2^ (2) = 7.25, *p* = 0.026, Figure 2): individuals with ASD identify a negative emotion more frequently than TD participants (326 vs. 280 for ASD and TD, respectively), whereas TD individuals identified a neutral emotion more frequently than ASD participants (182 vs. 129 for TD and ASD, respectively). The frequency of positive emotions was almost the same in the two groups (124 vs. 136 for TD and ASD, respectively).

For each group of words, an additional analysis was conducted only on those terms that were used at least five times by the two groups of participants.

**Figure 3:**
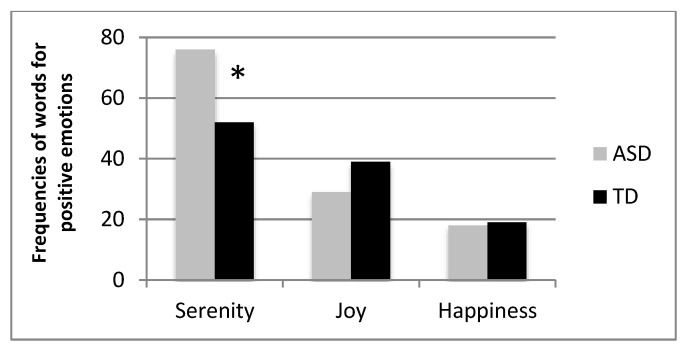
Frequency of use of terms corresponding to positive emotions distinguished by ASD and TD participants. Asterisks represent statistically significant effects.

**Figure 4:**
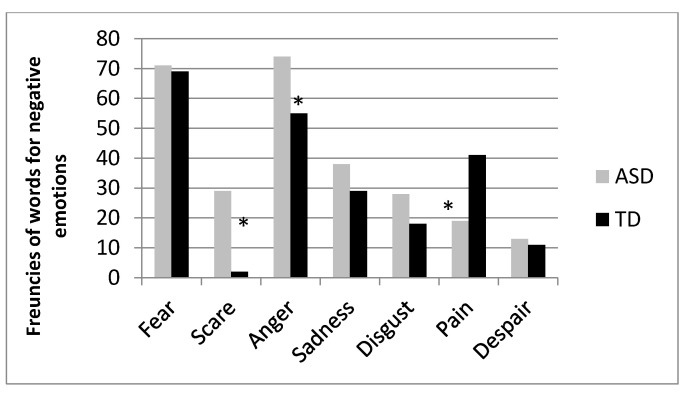
Frequency of use of terms corresponding to negative emotions distinguished by ASD and TD participants. Asterisks represent statistically significant effects.

**Figure 5:**
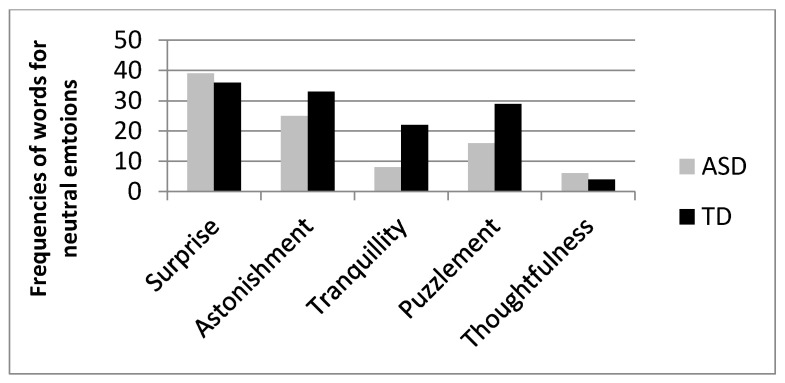
Frequency of use of terms corresponding to neutral emotions distinguished by ASD and TD participants.

For positive emotions (Figure 3), ASDs chose more often than TDs the word “serenity” (76 vs. 52 for ASDs and TDs, respectively, χ^2^ (2) = 4.99, *p* = 0.02) as compared to joy (29 vs. 39 for ASDs and TDs, respectively) and happiness (18 vs. 19 for ASDs and TDs, respectively); for negative emotions (Figure 4) ASDs used more often than TD the words “scare” (29 vs. 2 for ASDs and TDs, respectively, χ^2^ (6) = 33.861, *p* < 0.001) and “anger” (74 vs. 55 for ASDs and TDs, respectively), while TDs used more often the word “pain” (19 vs. 41 for ASDs and TDs, respectively). No statistical difference emerged for words denoting neutral affective states (Figure 5, χ^2^ (4) = 7.047, *p* > 0.05).

#### 3.1.3. Missing Values

**Figure 6:**
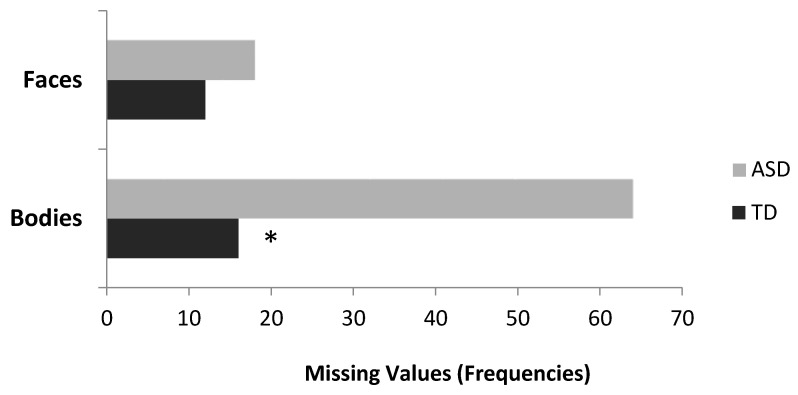
Frequencies of missing values for bodiless heads and headless bodies distinguished by ASD and TD participants. The asterisk represents a statistically significant effect.

Globally the number of missing values summed up to 110 (Figure 6). As expected, ASD group found more difficult to spontaneously associate an emotion to a stimulus (28 vs. 82 missing values for TD and ASD, respectively), but only when the stimulus was a body (bodies: 16 vs. 64 missing values for TD and ASD, respectively; heads: 12 vs. 18 for TD and ASD, respectively, χ^2^ (1) = 4.59, *p* = 0.03).

**Figure 7:**
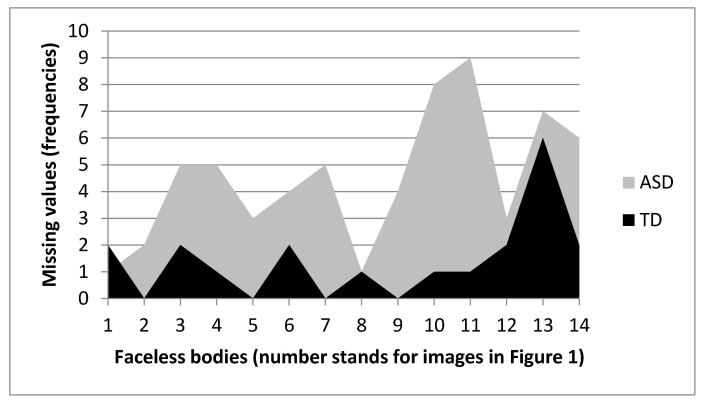
Frequencies of missing values for headless bodies for ASD and TD.

A qualitative inspection of the missing values for each stimulus (Figure 7) shows that for headless bodies stimuli with more missing values were 10b, 11b, 13b, and 14b. Missing values for bodies 10b and 11b were almost all due to ASD (for 10b: 8 vs. 1 for ASD and TD, respectively; for 11b: 9 vs. 1 for ASD and TD, respectively), whereas stimulus 13b was basically equally difficult for both groups (6 vs. 7 for ASD and TD, respectively).

#### 3.1.4. Emotions Attributed to Stimuli Using the List Provided

**Figure 8:**
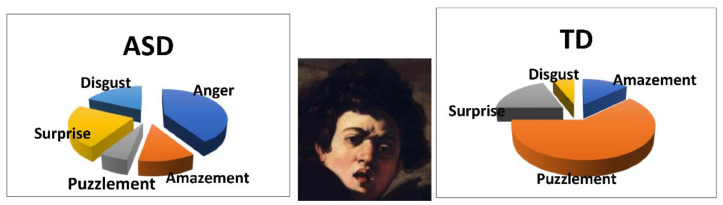
Different emotions attributed to image 4a (in the middle) by ASDs (**left**) and TDs (**right**).

**Figure 9:**
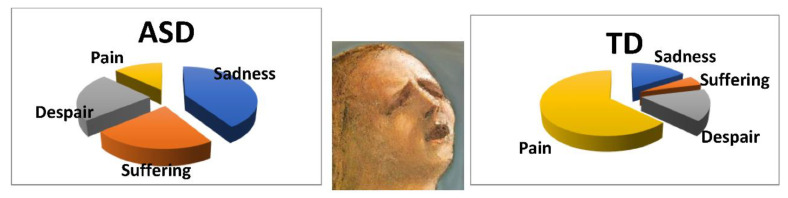
Different emotions attributed to image 9a (in the middle) by ASDs (**left**) and TDs (**right**).

We ran a chi-square test on the different emotions chosen by the two groups (list B) to describe the affective state conveyed by each stimulus. A statistical difference emerged for two bodiless heads: Caravaggio’s *Boy bitten by a lizard* (stimulus 4.a) and Masaccio’s *Eve* (stimulus 9.a). With regards to stimulus 4.a, ASDs reported anger (i.e., a negative emotion, Figure 8) as the main emotion conveyed by face, whereas TDs reported puzzlement (a neutral emotion) for the same stimulus, (χ^2^ (12) = 23.79, *p* = 0.022). With regards to 9.a both ASD and TD participants identified a feeling of suffering, but they used two distinct verbal labels with different intensity (respectively, “sadness” and “pain”, Figure 9, (χ^2^ (12) = 24.82, *p* = 0.035).

#### 3.1.5. Headless Body and Bodiless Head of a Same Artwork

For each single artwork we compared the emotions associated to bodiless heads and the corresponding headless bodies (list B), to test the congruency between the affective terms chosen to describe the two corresponding images.

Significant results are reported in Table 1, along with the emotion that scored the highest frequency for each stimulus. No effect of group was found.

### 3.2. Dynamicity Ratings

A mixed ANOVA with Stimulus Type (head vs. body) and Artwork (14 levels) as within-subjects factors and Group as between-subjects factor on dynamicity ratings revealed a significant main effect of Stimulus Type (F (1,43) = 28.062, *p* < 0.001) and Artwork (F (13,31) = 40.614, *p* < 0.001) and of the interactions Stimulus Type × Artwork (F (1,31) = 18.712, *p* < 0.001) and Artwork × Group (F (1,31) = 3.452, *p* = 0.002).

The dynamicity attributed to headless bodies (mean = 4.363, SE = 0.121) was higher than the dynamicity attributed to bodiless heads (mean = 3.785 SE = 0.174), with different artworks scoring, as obvious, different degrees of dynamicity. More interestingly, the interaction Stimulus Type × Artwork revealed that for some artworks the body was more dynamic than the face (Table 2), whereas for some others the face was perceived as more dynamic than the body (Table 3). In Table 2 and Table 3 the value of post-hoc t-tests are reported.

**Figure 10:**
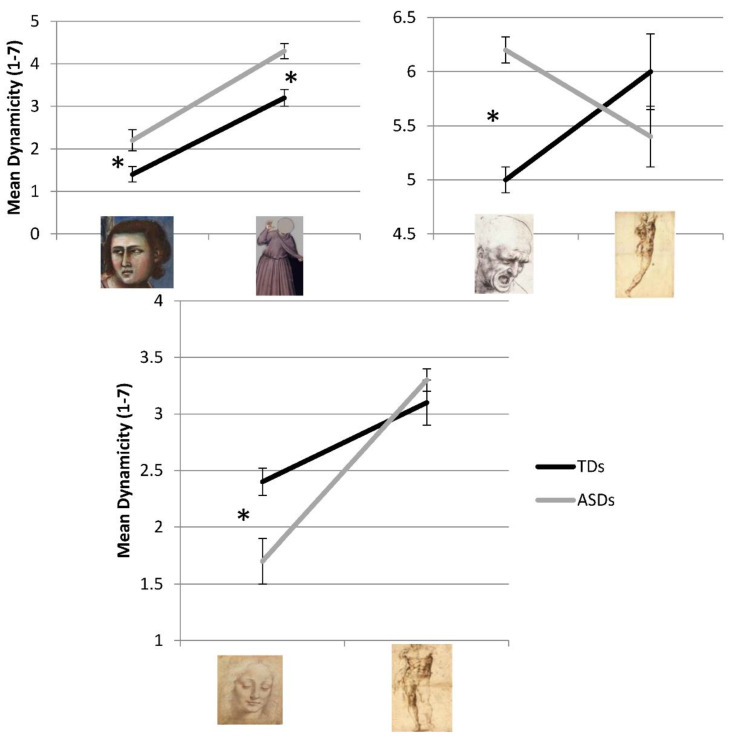
Level of dynamicity perceived by the two groups in bodiless heads (left) and headless bodies (right) for images 12a,b (upper left panel), 13a,b (upper right panel) and 14a,b (lower panel), respectively. Asterisks highlight significantly different means.

Regarding the interaction Artwork × Group, post-hoc t-tests proved significant for artworks 12, 13 and 14 (Figure 10). In particular, ASDs gave a higher value to the dynamicity of both head (t(20) = 2.682, *p* = 0.014) and body (t(20) = 1.919, *p* = 0.045) of image 12, and to the head of image 13a (t(20) = 1.919, *p* = 0.045), while they gave a lower score to the dynamicity of the head of image 14a (t(20) = 2.064, *p* = 0.048).

## 4. Discussion

We investigated whether individuals with autism spectrum disorders (ASD) perceive emotions portrayed in static artworks in the same way as typically developed individuals (TD). In particular, our hypothesis is focused on the relationship between motion and emotional expression. We hypothesized that all emotions can be classified in terms of inherent dynamism on a static-dynamic scale. According to our hypothesis, the more “dynamic” emotions (such as anger and fear, i.e., “e-motions”, [11]) “imply” motion, allowing the observer to extract dynamic information from such static representations. Given the well documented impairment of ASDs in both emotion recognition (e.g., [15,16,17]) and motion processing (due to their difficulty in integrating local elements of the input, [18]), we expected ASDs to be less accurate—or at least different—than TDs in recognizing portrayed emotions when these emotions are more “motion related” (i.e., e-motions), with a particular impairment for ‘emotional’ bodies. To test our hypothesis, we replicated with ASD individuals a study originally conducted with TDs [11], showing to both ASD and TD participants 14 bodiless heads and 14 headless bodies (taken from eleven static artworks and four drawings), asking them to associate an emotion to each.

Given the social impairment of our ASD sample, we had to make a change in the original procedure, which in turn allowed us to observe eventual differences in the range of verbal expressions defining emotions in the two groups of participants.

Contrary to our expectations, we found a similar range of verbal expressions defining emotions with both ASDs and TDs. It should be noticed that ASD individuals who participated in the present study are all high functioning adults. Given that ASD individuals are usually trained to recognize different emotions through faces, this, together with their high IQ (mean 121.059) could have positively influenced their range of verbal expressions for defining emotional and affective states. This result, although in contradiction with [42] is in agreement with studies with high functioning ASD children and adolescents, who show a good capacity to verbally express emotions (e.g., [52]).

However, this wide range of verbal expressions for defining emotions does not prove to be always effective. A difference is found in the ability to spontaneously assign an emotion to a headless body, and ASDs used more frequently than TDs words denoting negative emotions (for both faces and bodies) as compared to neutral emotions, which in turn are more frequently used by TDs.

A difference emerged between the two groups in the ability to associate an emotion to a headless body, with ASD individuals showing more missing values than TDs. This result supports the hypothesis according to which ASD individuals are particularly impaired in recognizing emotions from signals conveyed by the body (as compared to signals conveyed by the face), a hypothesis consistent with the WCC theory [20] and with ASD’s impairment to integrate details into a global vision [39,53]. The detail-based analysis used by ASD individuals was particularly evident during the whole experiment. The four images with the higher numbers of missing values are the *The Lamentation* by Nicolò dell’Arca (images 10a,b), the *Martyrdom of Saint Matthew* by Caravaggio (image 11a,b), and the two *Nude Males* by Michelangelo (images 13b and 14b). In all these images the body is incomplete (i.e., is not fully depicted). Several ASD participants were repeatedly complaining about the fact that, in image 11b “it is impossible to know what the boy is doing with the other arm (i.e., the one non-depicted)” and that, in the two nude males, “no arms are depicted and there are too many muscles. They are clearly anatomical studies with no emotional content.” (This is true, of course, but in saying this ASDs were showing their inability to consider neutral emotions, such as serenity or boredom, as such). On the contrary, TDs were easily answering by simply following the reasoning (often reported aloud) that if the man (for example for the study of image 14b) looks as if he is taking a stroll, he is “peaceful and quiet” or (for the study of image 13b) by deciding that “he is probably jumping for joy” (whereas ASDs kept on underlining how the body was tensed and slender). For the body of image 10b, the majority of ASDs were underlining the presence of the flowing drapery of the depicted figure by commenting “the dress is rippling as if there is a strong wind”, whereas TDs were assuming the same flowing drapery as due to a quick movement of the whole figure, which in turn should be a sign of anger or fear. Thus, the difference found between the two groups of participants in the number of missing values for headless bodies, together with spontaneous participants’ comments, supports the idea for which TDs apply what Zeki [54] calls “resolution of ambiguity” by relying on a combination of perceptual signals, expertise, and memories of personal emotional experiences. ASDs appear instead unable to complete what is not visible (or simply implied by an emotional expression). The presence of this impairment is not only consistent with the WCC theory [20] but also, more in general, with the Theory of Mind [55,56,57], which underlines the importance of the representation of other people’s mental states by interpreting perceptual cues of both their appearance (i.e., face, gaze, body posture, and so on) and behavior.

Incidentally, it should be noted that ASDs have proved to be unable to solve the ambiguity issue also in another category of perceptual ambiguous stimuli, specifically that of visual illusions [58,59,60,61]. Visual Illusions are by definition a category of perceptual phenomena that challenge concepts such as reality (in the sense of veridicality) and representation [62]. In this perspective the connection between visual illusions and the visual arts could be seen in the cognitive processes that “solve” the ambiguity in a way that is more (in the case of visual illusions) or less (in the case of the visual arts) stimulus driven, but that should definitely be solved in favor of a “non-veridical” (or at least “not necessarily veridical”) outcome. This possible lack of veridicality seems to be particularly difficult to overcome by ASDs.

It should also be noticed that adults with ASD are usually trained to recognize different emotions through faces, while they do not undergo any specific training for recognizing emotions through body postures. For this reason they could learn to compensate for a general deficit in emotion recognition, by learning associations over time between faces and emotional words, but they may not have a sufficiently deep understanding of emotions to generalize this association to unfamiliar stimuli, such as body postures [58]. We think that this association is particularly difficult for them because it implies an identification and interpretation of kinematic cues within a static representation (i.e., an inherent dynamism), as well as the temporal integration of details of the body with details of the corresponding faces. However, this result should be carefully considered, given the artistic nature of our stimuli, which makes the interpretation of the headless bodies difficult for both groups of participants. Indeed, when asked to freely associate an emotion to a headless body, both groups often report to find this task extremely difficult, supporting the idea that, regarding the emotions conveyed by static pictures, the emotion conveyed by the body is somehow secondary to the emotion conveyed by the face (i.e., body has a contextual effect, [63,64]). When this was the case, TDs reported neutral affective states for the corresponding “emotional body”, whereas ASDs were unable to give a spontaneous response. Even though we think that this difficulty is due to their impairment in recognizing emotions when conveyed by bodily kinematics (a difficulty which becomes more evident when the motion is only ‘implied’), the additional effect due to ASD’s difficulty in recognizing a neutral affective state [47] should be considered. This difficulty could also explain both the higher number of negative emotions spontaneously referred by ASDs and the difference found between the two groups in the interpretation of some stimuli.

Indeed, in the analysis of words spontaneously used by participants to describe emotions depicted in the stimuli, we found that ASDs often refer to emotional stimuli with negative words (such as anger or fear), while TDs refer to the same stimuli with words denoting neutral affective states (such as puzzlement). This result could depend not only on ASDs difficulty in recognizing neutral affective states [47], but also on the fact that they are usually trained to recognize basic emotions [1] and not neutral affective states. Thus, given that the task was to associate an emotion to each stimulus, it is possible that ASDs simply answer with a negative emotion, not considering a neutral affective state as an emotion. Another possibility is that ASDs overinterpret the negative component of emotive expressions in artistic images. These two possibilities could also interact: having to choose an emotion, ASDs exclude neutral affective states (because they do not consider them as emotions) *and* overinterpret the negative component of the emotional stimulus. This could explain not only the majority of negative words spontaneously used by ASDs, but also the fact that they interpret as “angry” the face of stimulus 4 (i.e., the boy bitten by a lizard by Caravaggio), whereas TDs interpret the same stimulus as “puzzled”. In other words ASDs, overinterpreting emotive signals, see anger where TDs simply see puzzlement, thus changing the whole meaning of the artwork (both groups attributed “disgust” to the body in image 4, which implies a different type of contrast with the emotion attributed to the face by TDs and ASDs, respectively).

However, this is true only for the negative component of emotional signals. Regarding positive emotions ASDs showed, as expected, a behavior substantially similar to that of TDs, confirming results showing that for ASDs happiness (and positive emotions in general) is the emotion better recognized [65,66,67,68] both in faces and in bodies [21].

In this perspective it is important to underline once again the artistic nature of our stimuli, for which we cannot say anything about “recognition of emotions”. Instead, with this kind of stimuli we should talk about different emotional interpretations of the same stimulus. Indeed, the results reported here should be read within the framework of static artworks, for which there are no previous works with ASD individuals. In this perspective the previous research by [11] could be seen as a sort of manipulation check, meant to establish which emotion corresponds to each face and body of the 14 different artworks used in this study. This check was necessary, given that no database is available for the emotional content of artworks. However, data from [11] are based on TD’s judgments, with no previous data available for ASDs. Thus, the fact that the emotion portrayed in the face of image 4a by Caravaggio is reported as puzzlement by the majority of TD participants (both in [11] and in the present study), does not necessarily mean that this face could not be seen as conveying different affective states (and in fact TDs also report amazement and surprise for the same image, and of course we do not know what emotion Caravaggio intended to depict on the face of the boy). In other words when ASDs report anger (instead of puzzlement) for the same stimulus, we cannot say that they do not recognize the neutral emotion of puzzlement, but instead that they do prefer to underline a negative component (which is probably present) of the emotional face, whereas TDs chose to underline a neutral one. This supposed overinterpretation of the negative component of the artistic emotional stimulus could also be found in the general behavior of our ASDs participants: on several occasions, to describe the emotion conveyed by the stimulus, they chose a word that, at least in Italian, underlines the violent or explosive component of the emotion (such as anger and scare), whereas TDs preferred to use words more related with feelings (such as pain). On the other hand, when using positive words, ASDs prefer to use a “softer” word, such as serenity, whereas TDs preferred a word underlining the dynamic component, such as joy.

Although some of these observations might be interesting (and nicely fitting with an analysis on emotions’ dynamicity), we think that these data should be better understood after a linguistic comparison on different “labels” used to indicate different affective states, with a particular stress on cultural differences (e.g., English vs. Italian). Indeed, this study was conducted with an Italian sample, and the English translation of some words used for different emotions is inevitably misleading. We are actually working on a project with different ethnic groups (i.e., Italian, Russian, and English) to better understand these possible differences, also in relation to inherent dynamism [13].

Regarding the emotions conveyed by the bodiless heads and headless bodies of the same character, the differences that emerged are mainly related to different artworks, with no difference between the two groups of participants (the only interesting observation applies to images 4a,b, with the majority of ASDs interpreting the face as “angry”, but interpreting the body as “disgusted”, a result already discussed above). A similar result was found for the difference in the degree of dynamicity assigned to face and body of the same artwork. A discussion of these differences would be too speculative at this stage, although they might lead us in formulating hypotheses that will be tested in future works. What is interesting in these differences is the contrast between opposite emotions conveyed by the face and the body of the same character, combined with different—or similar—degrees of inherent dynamism. For example, in images 2a,b (the *Orphan girl* by Delacroix) the head of the girl is turned away from the observer (i.e., with implied motion), while the body is portrayed as sitting (i.e., static), facing the observer. This apparent contrast in the implied motion of the face on one hand and of the body on the other is mirrored in participants’ judgments, who scored the dynamicity of the face higher than that of the body and attributed to the face emotions as surprise and fear (i.e., e-motions), while they attributed to the body mainly puzzlement and serenity (which in turn could be considered as “static” emotions). These observations should be better clarified with further studies, in which the posture of the face (i.e., full face, three quarter profiles, head slightly tilted and so on) of the portrayed character, as well as the posture of the body, should be carefully evaluated when choosing artworks as stimuli. In the present study this control was not made, given that we chose a number of artworks, which, according to our hypothesis, could be classified in terms of emotions *and* dynamism, with the only constraints of limiting our analysis to figurative artworks.

As far as dynamicity is concerned, both groups assigned a higher value of dynamicity to the headless bodies as compared to the bodiless heads, an effect consistent with our previous findings [11] and generally expected.

Contrary to our expectations, overall no difference emerged between the two groups, but the interaction Artwork × Group showed that, at least for some images, ASDs assigned a different value than TDs to perceived dynamism. In particular those images are images 12a,b, 13a,b and 14a,b.

Regarding images 12a,b (in which both head and body were taken from *The expulsion of merchants from the temple* by Giotto), both groups of participants gave a higher score to the dynamicity of the face compared to the dynamicity of the body (a difference which is overall significant), but ASDs gave higher scores than TDs to the dynamicity of both face and body of the portrayed character. We could speculate that the overvaluation of the dynamicity of a figure that appears as basically static could be due to an excessive importance given by ASDs to details as the head slightly tilted and the body slightly slanted. Another argument should be made for images 13a,b and 14a,b which, as said in the methods section, were conceived as a control for our hypothesis. In those images headless bodies and bodiless heads are not taken from the same artworks. In particular heads were taken from masterpieces by Leonardo Da Vinci, while bodies were authored by Michelangelo Buonarroti. A qualitative analysis of ASDs’ data for those artworks is quite informative on their different way of interpreting the visual arts. In both cases the difference between the group is due to the face of the portrayed character: in image 13a ASDs give a higher dynamicity value to the face as compared to TDs, while in image 14a they give a lower score to the dynamicity of the face as compared to TDs. It should be noticed that in images 13a,b this difference caused an “inversion” to the dynamicity attributed to the face and body, with head evaluated as more dynamic than body by ASDs and as less dynamic than body by TDs. This result confirms both the general tendency of ASDs to focus on faces and the fact that they give a higher importance to “dynamic” details as the mouth open in the face of image 13a. In images 14a,b instead both ASDs and TDs gave a lower score to the face as compared to the body, but for ASDs this difference was higher. We think this result is particularly interesting, given that it shows that the tendency of ASDs to evaluate the stimulus in every single detail, independently from the task, could lead them to enhance the difference between the face and the body, which in fact, in this case, were actually taken from different artworks. In other words, even though the task was to evaluate the dynamicity of the figure, ASD participants were unable to consider dynamicity per se, including in the evaluation also details which could be better considered as indexes of different artistic style (e.g., traits typical of Leonardo’s or Michelangelo’s style). In this particular case they were, so to say, “right”, given that heads and bodies in images 13a,b and 14a,b were in fact authored by different artists, whereas in TDs’ judgments those differences are flattened.

As stated in the introduction, we think that the emotional content of the characters portrayed could differently contribute to the inherent dynamicity of the whole artworks. Thus, our hypothesis was ultimately based on a sort of “contextual effect”, where the emotions conveyed by the face may either enhance or contrast the emotion conveyed by the body, and the final dynamic/emotional content of the whole portrayed figure may in turn either enhance or contrast the global emotional and dynamical impression of the whole artwork.

With the present research we should revise our general hypothesis, at least for ASDs. Their difficulty in associating emotions to bodies put in evidence that their hypersensitivity to local elements of the overall visual picture made them less susceptible to the effect of context, which in turn could be translated in a different way of appreciating the visual artworks in general. Even though at this stage this remains a matter of speculation, with the present research we understood that this contextual effect is more complex than expected, being also associated with verbal, as well as categorical, abilities.

We suppose that visual perception focused on local information is used as a compensatory strategy for ASD individuals. For this reason it is more difficult for them to identify emotions in bodies, which may be lacking relevant details that could uncover intentions and emotions out of the context. Despite the similarities, we suggest examining in the future the perception of body emotions in the visual arts and to study differences in terms of verbal labels used to name affective states. This could help to highlight a possible different cognitive style in appreciating the visual arts by individuals with ASDs (as well as by possibly other groups of individuals), especially when the emotional content is considered.

## 5. Conclusions

The present study is the first attempt to test how ASDs interpret visual artworks in terms of their emotional content. Starting from the hypothesis that all emotions can be classified in terms of inherent dynamism on a static-dynamic scale, we expected ASDs to be less accurate—or at least different—than TDs in recognizing portrayed emotions when these emotions are more motion related (i.e., e-motions), with a particular impairment for ‘emotional’ bodies. Consequently, we also expected to find a difference between the two groups in the correspondence between the emotion attributed to the face and the corresponding body emotion. As expected, ASDs found more difficult to spontaneously associate an emotion to headless bodies than TDs, although no difference emerged regarding e-motions. The overall value of inherent dynamicity assigned to each emotion was not different between the two groups, but some interesting differences were found in some specific artworks.

The possibility we gave to our participants to freely associate each stimulus to an emotion, or an affective state, turned out to have some positive, as well as negative, consequences. Regarding the first, this procedure allowed us to observe the range of verbal expressions defining emotions between the two groups, as well as to have a better qualitative description of ASDs’ overall “emotional” experience. We thus pointed out that, although ASDs and TDs use broadly the same range of words for emotions, ASDs refer more often to negative emotions, whereas TDs are more inclined to recognize neutral affective states, with some interesting differences in the choice of words corresponding to a specific emotion. This difference could be due either to an overinterpretation of negative aspects associated to emotions by ASDs, or, alternatively, to the fact that they are usually trained to recognize basic emotions [1] but not neutral affective states, and for this reason they do not consider them as emotions.

Another positive consequence of our procedure is that the higher number of missing values for bodies in ASDs highlights not only the higher difficulty of the task, but also their inability to “guess” or, in other words, to “solve the ambiguity” [54], which is in fact what makes the artistic fruition so “personal” and peculiar. Indeed, even though both groups reported a higher difficulty to associate an emotion to a body, TDs try to guess (solving the ambiguity in a personal way), whereas ASDs prefer not to answer. In our view, this different behaviour highlights a different approach towards the artistic experience, possibly less emotional and more “rationale”, which deserves further investigation.

However, the high number of missing values for ASDs in the headless bodies was also a negative consequence of our procedure, because it does not allow us to draw any definite conclusion regarding their ability to read e-motions in portrayed characters. As a negative consequence of our procedure, we should also consider the fact that the wide range of verbal expressions spontaneously produced to define emotions (in both groups) caused a dispersion of data on a too small sample, a problem that should be considered when testing clinical populations.

Altogether our data pointed out the necessity of devising a new methodological approach to understand different cognitive styles characterizing the aesthetical/emotional experience of artworks. Our results, as well as the qualitative analysis of ASDs behavior when asked to report the emotional content of artistic characters, suggest that individuals with ASDs may perceive the visual artworks in a different way. Given that this difference emerged only in some artworks, we suggest to carefully consider the use of artworks as stimuli for future studies and possibly to prepare a database for artworks classified in terms of emotions they portrayed. We are currently working on the creation of such a database.

In conclusion, we think that the study of ASDs’ way of experiencing the visual arts is not only an opportunity for future research on ASD, but also a useful tool towards a better understanding of different ways of perceiving and experiencing reality, being the visual arts a representation of a personal (i.e., of the artist) take on reality.

## Figures and Tables

**Table 1:** Different emotions identified for face and body of the same static artwork.

Stimuli	Chi-square	Head	Body
**2**	χ^2^ = 212.73, *p* < 0.001	Surprise, Astonishment	Serenity, Tranquillity
**4**	χ^2^ = 225. 36, *p* = 0.002	Puzzlement	Disgust
**6**	χ^2^ = 301.9, *p* < 0.001	Puzzlement	Joy, Serenity
**7**	χ^2^ = 159.47, *p* = 0.52	Surprise, Astonishment	Fear
**10**	χ^2^ = 277.04, *p* = 0.006	Fear	Anger
**11**	χ^2^ = 214.01, *p* = 0.026	Astonishment	Fear
**13**	χ^2^ = 149.352, *p* = 0.007	Anger	Joy

**Table 2:** Post-hoc t-tests for images with different dynamicity, headless bodies (B) more dynamic than bodiless heads (A).

Images (a < b)	Post-hoc t-test
1	t(44) = 4.268 *p* < 0.0001
4	t(44) = 4.354 *p* < 0.0001
5	t(44) = 7.416 *p* < 0.0001
6	t(44) = 7.543 *p* < 0.0001
7	t(44) = 2.578 *p* = 0.013
10	t(44) = 2.708 *p* = 0.010
12	t(44) = 9.949 *p* < 0.0001

**Table 3:** Post-hoc t-tests for images with different dynamicity, bodiless heads (A) more dynamic than headless bodies (B).

Images (a > b)	Post-hoc t-test
2	t(44) = 6.299 *p* < 0.0001
9	t(44) = 3.212 *p* = 0.002
13	t(44) = 4.619 *p* < 0.0001
